# Biological properties of self-assembled nanofibers of elastin-like block polypeptides for tissue-engineered vascular grafts: platelet inhibition, endothelial cell activation and smooth muscle cell maintenance

**DOI:** 10.1093/rb/rbac111

**Published:** 2022-12-28

**Authors:** Kazuki Natsume, Jin Nakamura, Kazuhide Sato, Chikara Ohtsuki, Ayae Sugawara-Narutaki

**Affiliations:** Department of Materials Chemistry, Graduate School of Engineering, Nagoya University, Nagoya 464-8603, Japan; Department of Biological Functions Engineering, Graduate School of Life Science and Systems Engineering, Kyushu Institute of Technology, Fukuoka 808-0196, Japan; Institute for Advanced Research, Nagoya University, Nagoya 464-8601, Japan; Department of Respiratory Medicine, Graduate School of Medicine, Nagoya University, Nagoya 466-8560, Japan; Department of Materials Chemistry, Graduate School of Engineering, Nagoya University, Nagoya 464-8603, Japan; Department of Energy Engineering, Graduate School of Engineering, Nagoya University, Nagoya 464-8603, Japan

**Keywords:** elastin-like polypeptide, nanofiber, platelet, endothelial cell, smooth muscle cell

## Abstract

Strategic materials design is essential for the development of small-diameter, tissue-engineered vascular grafts. Self-assembled nanofibers of elastin-like polypeptides represent promising vascular graft components as they replicate the organized elastin structure of native blood vessels. Further, the bioactivity of nanofibers can be modified by the addition of functional peptide motifs. In the present study, we describe the development of a novel nanofiber-forming elastin-like polypeptide (ELP) with an arginine–glutamic acid–aspartic acid–valine (REDV) sequence. The biological characteristics of the REDV-modified ELP nanofibers relevant to applications in vascular grafting were compared to ELP without ligands for integrin, ELP with arginine–glycine–aspartic acid (RGD) sequence, collagen and cell culture glass. Among them, REDV-modified ELP nanofibers met the preferred biological properties for vascular graft materials, i.e. (i) inhibition of platelet adhesion and activation, (ii) endothelial cell adhesion and proliferation and (iii) maintenance of smooth muscle cells in a contractile phenotype to prevent cell overgrowth. The results indicate that REDV-modified ELP nanofibers represent promising candidates for the further development of small-diameter vascular grafts.

## Graphical abstract

**Figure:**
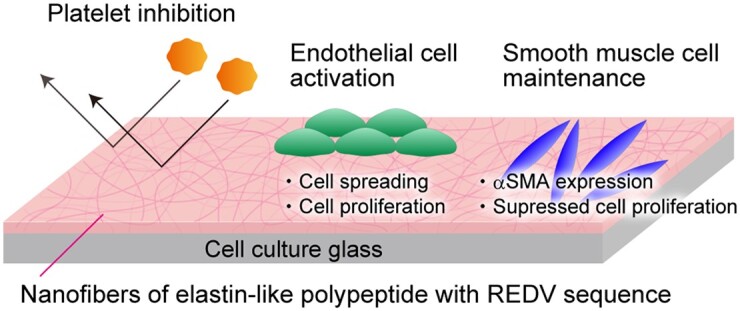

## Introduction

Currently available synthetic vascular grafts, which are composed of expanded polytetrafluoroethylene or poly(ethylene terephthalate), represent a useful alternative to large diameter (>6 mm) blood vessels. However, they are not suitable for smaller-diameter applications due to loss of patency over time [1–8]. Tissue-engineered vascular grafts (TEVGs) that are remodeled *in vivo* from artificial grafts to autologous vascular tissues may overcome the limitations of conventional grafts [1–8]. An ideal TEVG should meet the following requirements: biocompatibility, non-thrombogenicity, durability and compliance comparable to native vessels, suture integrity and controlled biodegradability. Considerable work has been conducted in the development of cell-seeded grafts as they better mimic native vessels that possess the ideal properties for grafting [1–4]. Recently, cell-free TEVGs have attracted increasing attention due to their cost-effectiveness and off-the-shelf availability, which are important aspects for clinical use [5–8]. The design of strategic materials based on advanced polymer/biopolymer chemistry is essential for the development of cell-free TEVG.

In the pioneering work for producing cell-seeded TEVG, Weinberg and Bell [9] used collagen gel to support vascular cells (endothelial cells (ECs), smooth muscle cells (SMCs) and fibroblasts). The EC layer on the collagen gel functioned like the endothelium of a normal blood vessel. Despite its excellent cytocompatibility, the use of collagen in cell-free TEVG is limited because collagen can bind von Willebrand Factor (vWF) and blood coagulation proteins, which promotes platelet adhesion and thrombus formation [10, 11].

Elastin, an essential extracellular matrix protein within the arterial wall, is a promising candidate as a component of cell-free TEVGs [12–14]. The primary role of elastin is to provide the arterial wall with the deformability and resilience required to withstand pulsatile blood flow [15]. Elastin also functions in regulating the migration, proliferation and differentiation of SMCs, which have important implications in preventing intimal hyperplasia [16, 17]. Various forms of elastin including α-elastin [18], elastin from bovine neck ligament [19] and elastin-derived peptides [20] interact with ECs to promote proliferation. Moreover, the anti-thrombogenic properties of elastin have been widely recognized [21, 22]. These advantages make elastin an attractive material for constructing cell-free TEVGs. Chuang *et al.* [23] demonstrated that elastin-rich scaffolds, in which cells and collagen were removed by alkaline treatment from porcine carotid arteries followed by stabilization with a polyphenol, allowed cell infiltration into the scaffold and matrix remodeling *in vivo*. Elastin was combined with collagen [24, 25], polydioxanone [26] and collagen/biodegradable synthetic polymers [19, 27] to enhance the mechanical and biological properties of the tubular scaffold. The fabrication of elastin-containing, triple-layered vascular grafts that resemble native arterial architecture has also been described [28, 29]. However, the application of biologically-derived elastin has been limited by poor processability and substantial batch-to-batch variation. Calcification of elastin after implantation has also been observed in animal models [30].

Protein engineering provides alternate solutions to the use elastin-based materials in cell-free TEVGs. Weiss *et al.* [31] established the expression of recombinant human tropoelastin (rhTE), the soluble precursor of elastin. Synthetic grafts comprising crosslinked rhTE and polycaprolactone had equivalent mechanical properties to the human internal mammary artery in addition to enhanced EC interactions and reduced platelet attachment compared to polycaprolactone alone [32]. Even more strategic materials design is possible using elastin-like polypeptides (ELPs), a class of recombinant proteins that contain repetitive peptide motifs, such as VPGXG, VAPGVG or KAAK (V, valine; P, proline; G, glycine; A, alanine; K, lysine; and X, any amino acid except P), found in natural elastin. Repetitions of VPGXG and VAPGVG are found in the hydrophobic domain of tropoelastin and contribute to elasticity [33] and interactions with vascular cells [34, 35], respectively. KAAK peptide motifs, which are present in the crosslinking domain of tropoelastin, can be crosslinked with each other in the presence of amine-reactive crosslinkers. Woodhouse *et al.* [36] demonstrated decreased platelet activation using an ELP comprising exons 20–24 of tropoelastin (designated ELP4 due to the presence of four crosslinking domains) to coat synthetic polymer catheters compared to uncoated controls. The anti-thrombotic effects and EC adhesion properties of ELP4 were maintained after covalent bonding to a material surface [37]. ELP4-coated materials also maintained SMC in a contractile phenotype [38]. Cell-binding peptide motifs, such as arginine–glycine–aspartic acid (RGD) and arginine–glutamic acid–aspartic acid–valine (REDV), have been incorporated into ELP sequences to increase interactions with ECs [39–42]. RGD is a universal cell-binding sequence while REDV is specifically recognized by the integrin α_4_β_1_ present in ECs. De Torre *et al.* [39, 40] developed hydrogels of ELP containing multiple RGD or REDV motifs. These hydrogels were fabricated by click reactions from azide- and alkyne-modified ELPs and used to cover nitinol (Ni-Ti) stents [39], with the proliferation of ECs found to be greater on stents covered by RGD- and REDV-containing hydrogels. RGD-modified click gels were combined with textile components to form vascular grafts of ∼2 mm diameter [41], with *in vitro* studies demonstrating that grafts with open pore structures increased cell ingrowth while maintaining non-thrombogenicity and mechanical performance. Mahara *et al.* [42] fabricated tubular scaffolds using RGD-modified, crosslinked ELP reinforced with electrospun poly(L-lactic acid). When tubular scaffolds with an inner diameter of 1 mm and length of 5 mm were implanted into the abdominal aortae of rats, connective tissue was found to grow along the scaffold luminal surface resulting in new vascular tissue within one month. The biodegradability of ELP can also be controlled by incorporating cleavage sites recognized by proteases [43], although this approach has yet to be applied to the fabrication of vascular grafts.

The fabrication of organized elastin structures in TEVGs is another important hurdle to the long-term success of vascular grafts [14]. In arteries, elastin is present as continuous nanofibers and layered nanosheets with both structural and cell-signaling roles [14]. However, the fabrication of complex nanoarchitectures using currently available ELPs remains technically challenging. Our group previously developed a novel class of ‘double-hydrophobic’ ELP block copolymers, termed GPG, which self-assemble to form nanofibers in water [44–49]. The block copolymer, comprising a (VGGVG)_5_-(VPGXG)_25_-(VGGVG)_5_ sequence, was designed by mimicking the uneven distribution of glycine-rich and proline-rich hydrophobic domains in tropoelastin [44]. At temperatures above 37°C, GPG assembles into nanoparticles by the hydrophobic collapse of (VPGXG)_25_ followed by polymerization into nanofibers through the formation of hydrogen bonds between (VGGVG)_5_ domains. As the nanofibers of GPG are more than 20 μm in length and can form network-like structures to create stable hydrogels [45, 46], GPG may have utility as components of TEVGs that mimic arterial elastin nanostructures. Furthermore, various peptide motifs, including KAAK [47], GRGDS [48] and silver-binding sequence [49], can be bound to the C-terminus of GPG to modify its physicochemical and biological characteristics without impairing its fiber-forming abilities.

In this article, we have synthesized a new derivative of GPG with GREDV peptide motif (GPG-REDV) with a fiber-forming ability (Table 1) and evaluated whether it meets the biological properties required for TEVG. GPG without ligand sequences, GPG with GRGDS motif (GPG-RGD), collagen and cell culture glass were used for comparison. The results showed that only GPG-REDV fulfilled all the preferred biological properties for TEVG materials, i.e. inhibition of platelet adhesion, EC proliferation and maintenance of SMCs in contractile phenotype.

**Table 1.** GPGs used in the present study

Sample name	Amino acid sequence	Molecular weight (Da)
GPG	MKL-(VGGVG)_5_-LWLGSG-[(VPGVG)_2_VPGFG(VPGVG)_2_]_5_-KL-(VGGVG)_5_-LWLEHHHHHH	16 781
GPG-RGD	MKL-(VGGVG)_5_-LWLGSG-[(VPGVG)_2_VPGFG(VPGVG)_2_]_5_-KL-(VGGVG)_5_-LWLEHHHHHH-KAAKGRGDS	17 670
GPG-REDV	MKL-(VGGVG)_5_-LWLGSG-[(VPGVG)_2_VPGFG(VPGVG)_2_]_5_-KL-(VGGVG)_5_-LWLEHHHHHH-KAAKGREDV	17 736

## Materials and methods

### Protein expression and purification

The construction of the pET22b(+)-GPG1 and pET22b(+)-GPG3 plasmids encoding GPG and GPG-RGD, respectively, has previously been reported [44, 48]. The construction of the pET-22b(+)-GPG-REDV plasmid encoding GPG-REDV is described in detail in the Supplementary Data. Plasmids were transformed into the *Escherichia coli* BLR(DE3) strain, which is suitable for expressing recombinant proteins with highly repeating motifs. All GPGs were expressed and purified as previously reported [44, 48]. Briefly, polypeptides were purified using Ni-NTA affnity chromatography as all contained His-tags at the C-terminus. Purification of GPGs was confirmed by sodium dodecyl sulfate–polyacrylamide gel electrophoresis (SDS-PAGE) and matrix-assisted laser desorption/ionization time-of-flight mass spectrometry (MALDI-TOF-MS) using an AXIMA-CFR Plus spectrometer (Shimadzu, Japan). Polypeptides were stored as lyophilized powder at –20°C prior to use.

### Nanofiber formation

Polypeptide powders were dissolved in chilled water and agitated at 4°C to obtain monomer solutions. Polypeptide concentrations were determined by measuring the absorbance at 280 nm using a Nanodrop 2000 UV-visible spectrometer (Thermo Fisher Scientific, USA). Adequate amounts of ultrapure water (conductivity: 18.2 MΩ) were added to adjust polypeptide concentrations to 20 μM (0.33, 0.35 and 0.35 mg/ml for GPG, GPG-RGD and GPG-REDV, respectively). The solutions were then incubated at 37°C for 1 week.

### Nanofiber coating

GPG nanofibers were coated onto substrates as previously described with minor modifications [48]. Nanofiber suspensions of GPGs were diluted to 0.1 mg/ml with ultrapure water at 37°C. The wells of 96-well borosilicate cell imaging plates (Eppendorf, Germany; the bottom area of the well is 0.3 cm^2^) were coated with 30 μl of GPG, GPG-RGD or GPG-REDV fiber suspensions and allowed to dry at 37°C for 4 h. After discarding the remaining solution, nanofiber-coated plates were further dried at 37°C for 12 h. Plates were then sterilized under ultraviolet light in a safety cabinet. Finally, each well was washed with 100 μl HEPES-buffered saline twice. Bovine Type I collagen (Sigma-Aldrich, USA) at 0.1 mg/ml was coated onto wells using the same procedure for comparison.

Eight-well cell imaging coverglasses (Eppendorf, Germany; the bottom area of the well is 0.9 cm^2^) were used instead of cell imaging plates where detachment of the coverglass was necessary. Wells were coated with 90 μl aliquots of samples.

### Scanning electron microscopy

Cell imaging coverglasses coated with samples were sputtered with osmium tetroxide at a thickness of 3 nm using an osmium coater (Osmium plasma coater OPC60A, Filgen, Japan). Surface morphologies were observed using a field-emission scanning electron microscope (FE-SEM, JSM-7500FA, JEOL, Japan) with an accelerating voltage of 2 kV.

### Water contact angle measurements

Ultrapure water (5 μl) was dropped on the protein-coated or non-coated cell imaging coverglasses. Images of water droplets were acquired with a USB MicroScope M2 (Scalar, Japan), and the contact angles were measured by using Image J software (National Institutes of Health, USA) from eight or more measurements.

### Platelet adhesion test

Platelet adhesion test was performed according to the literature [50]. Whole blood (2.7 ml × 2) was collected from a healthy human donor using blood collection tubes containing 3.2% sodium citrate buffer. The ratio of sodium citrate buffer to blood was 1:9. Platelet-rich plasma (PRP) was collected by centrifugation (200 × *g*, 5 min) of whole blood. Collected PRP was further centrifuged (1500 × *g*, 10 min) to obtain platelet-poor plasma (PPP). The numbers of platelets in PRP and PPP were counted using a phase contrast microscope. PRP and PPP were then mixed to a final platelet density of 1.75 × 10^7^ cells/ml. Platelet suspensions (200 μl) were added to protein-coated or non-coated cell imaging coverglasses and incubated at 37°C for 1 h. Each coverglass was washed twice with phosphate-buffered saline (PBS) and fixed with 1.0% glutaraldehyde in PBS at 37°C for 2 h. Coverglasses were washed sequentially with PBS, a 1:1 solution of PBS and water, and water. After drying at 37°C, coated coverglasses were detached from the chamber and sputter-coated with osmium tetroxide. The number of platelets adhered to the surface was counted using FE-SEM. Platelets are classified into three types according to the number of pseudopodia: Type 1, 0; Type 2, 1–2; and Type 3, more than 2. The number of platelets was counted manually from at least 40 views for each sample. This experiment was carried out with ethical approval (No. 2017-0534-3) by ethical review board in Nagoya University to use human whole blood.

### Cell culture

Human umbilical vein endothelial cells (HUVECs) and human umbilical arterial smooth muscle cells (HUASMCs) were purchased from PromoCell, Germany. HUVECs were maintained in Endothelial Cell Growth Medium 2 (PromoCell, Germany). HUASMCs were maintained in Smooth Muscle Cell Growth Medium 2 (PromoCell, Germany). Cells were cultured in a humidified atmosphere with 5% CO_2_ at 37°C. Cell passages 3–6 (HUVECs) and 4–5 (HUASMCs) were used for experiments.

### Cell adhesion

HUVECs or HUASMCs were seeded onto protein-coated or non-coated surfaces of 96-well borosilicate cell imaging plates at a density of 1.0 × 10^4^ cells/well with 100 µl cell culture medium. Cells were incubated at 37°C in a humidified atmosphere with 5% CO_2_. After 24 h, wells were rinsed with PBS to remove unattached cells. Samples were then fixed with 4% paraformaldehyde (Wako Pure Chemical Industries, Japan), washed with PBS and permeabilized with 0.5% Triton-X-100 (Wako Pure Chemical Industries, Japan) for 25 min. Nonspecific binding sites were blocked with 1% bovine serum albumin (BSA, Wako Pure Chemical Industries, Japan) for 1 h. Thereafter, cells were incubated overnight at 4°C with rabbit monoclonal anti-human vinculin antibody (Life Technologies, USA) in 1% BSA. Cells were stained with Alexa Fluor™ 546 goat anti-rabbit IgG (Life Technologies, USA) in 1% BSA for 1 h at room temperature, Alexa Fluor™ 488 phalloidin (Life Technologies, USA) in PBS for 30 min at 4°C and NucBlue™ Fixed Cell Stain ReadyProbes™ (Life Technologies, USA) in PBS for 5 min at room temperature. Fluorescent images were acquired using a fluorescence microscope (BZ-X710, KEYENCE, Japan). All experiments were performed in hexaplicate. Cell numbers and coverage were calculated using built-in Hybrid Cell Count software from 20 or more images.

### NO release

The release of nitric oxide (NO) from HUVECs was measured using OxiSelect™ In Vitro Nitric Oxide (Nitrite/Nitrate) Assay kits (Cell Biolabs, USA); a colorimetric assay that indirectly measures NO by ${\text{NO}}_{2}^{-}$/${\text{NO}}_{3}^{-}$ determination based on the extremely short half-life of NO. Briefly, HUVECs (1.0 × 10^4^ cells/well) were seeded and cultured for 24 h. ${\text{NO}}_{3}^{-}$ in the supernatant of cell culture medium were first converted to ${\text{NO}}_{2}^{-}$ by nitrate reductase enzyme. Total ${\text{NO}}_{2}^{-}$ was detected with Griess reagents as a colored azo dye product by measuring the absorbance at 540 nm using a microplate reader (Epoch 2, Biotek, Japan). The amount of NO was estimated using the calibration curves of standard samples. Results were calculated from four measurements.

### Cell proliferation

Cells were seeded and cultured according to the methods described above except for initial seeding densities of 1.0 × 10^3^ cells/well for HUVECs and 2.0 × 10^3^ cells/well for HUASMCs. After 1, 3, 5 or 7 days, cells were rinsed, fixed, blocked and subjected to immunostaining. HUVECs were incubated with vWF monoclonal antibody (F8/86, Life Technologies, USA) and HUASMCs were incubated with α-smooth muscle actin (αSMA) monoclonal antibody (Life Technologies, USA) in 1% BSA for overnight at 4°C. After washing with PBS, cells were stained with goat anti-mouse IgG (H + L) highly cross-adsorbed secondary antibody, Alexa Fluor™ 546 (Life Technologies, USA) in 1% BSA for 1 h. Then, actin and cell nuclei were stained as described above. All experiments were performed in hexaplicate. Cell numbers were counted from fluorescence microscopy images as described above.

### Reverse transcription–polymerase chain reaction

The mRNA expression levels of αSMA (gene: *Acta2*) were measured using the quantitative reverse transcription–polymerase chain reaction (qRT-PCR). HUASMCs (2.0 × 10^3^ cells/well) were cultured on protein-coated or uncoated surfaces for 7 days. RNA was extracted and reverse-transcribed using CellAmp™ Direct Lysis and RT (Takara Bio, Japan). Samples for qRT-PCR contained the reverse-transcribed reaction mixture (4 μl), forward and reverse primers (0.4 μM each, Takara Bio, Japan) and 1 × TB Green Premix Ex Taq II (Takara Bio, Japan). qRT-PCR was performed using a Thermal Cycler Dice^®^ Real Time System III (Takara Bio, Japan) with TB Green^®^ probes (Takara Bio, Japan) for *Acta2* using the following conditions: initial hold at 95°C for 30 s; 40 cycles of 95°C for 5 s and 60°C for 30 s; and 1 cycle of 95°C for 15 s, 60°C for 30 s and 95°C for 15 s. The relative expression levels of target genes were calculated according to the ΔΔCt method using glyceraldehyde-3-phosphate dehydrogenase (GAPDH) as an internal reference. Primers were synthesized by Takara Bio, Japan. Primer sequences were as follows: Acta2, forward 5′-ATT GCC GAC CGA ATG CAG A-3′, reverse 5′-ATG GAG CCA CCG ATC CAG AC-3′; GAPDH, forward 5′-GCA CCG TCA AGG CTG AGA AC-3′, reverse 5′-TGG TGA AGA CGC CAG TGG A-3′.

### Statistical analyses

Data are expressed as mean ± standard error. Statistical analyses were performed using the unpaired two-way Student’s *T*-test. *P*-values <0.05 were considered statistically significant.

## Results

### Characterization of protein-coated surfaces

The successful synthesis and the fiber-forming ability of GPG-REDV were confirmed (Supplementary Figs S1 and S2), as previously demonstrated for GPG and GPG-RGD [48]. GPG, GPG-RGD and GPG-REDV nanofibers as well as collagen were coated onto borosilicate cell imaging plates and allowed to dry.

**Figure 1.**
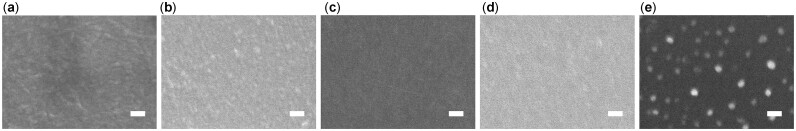
FE-SEM Images of (**a–d**) protein-coated and (**e**) uncoated coverglasses. Coverglasses coated with nanofibers of (**a**) GPG, (**b**) GPG-RGD, (**c**) GPG-REDV and (**d**) collagen. Scale bars correspond to 100 nm.

FE-SEM images of sample-coated and uncoated coverglasses are shown in Fig. 1. There were particulate objects of <50 nm in size on the uncoated coverglasses provided by the manufacturer (Fig. 1e). These particles were not observed after sample coating, indicating proteins were successfully deposited on sample substrates.

Water contact angle measurements were performed to evaluate the surface wettability of sample-coated and uncoated coverglasses (Table 2). Uncoated glass had the highest contact angle of 76.7 ± 1.7°. Decreases in contact angles were observed after sample coating, indicating increased hydrophilicity. The water contact angles for GPG, GPG-RGD and GPG-REDV nanofibers were equivalent, while collagen had the lowest water contact angle.

**Table 2.** Water contact angles on various surfaces

Sample	GPG	GPG-RGD	GPG-REDV	Collagen	Glass
Representative image	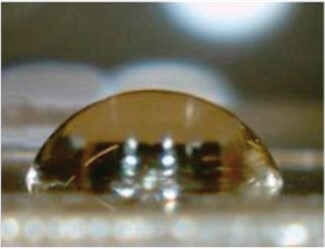	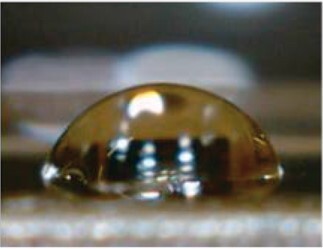	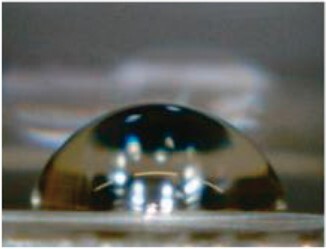	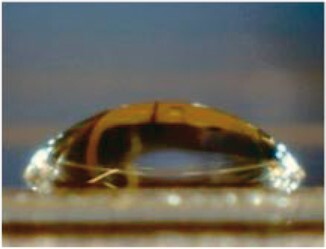	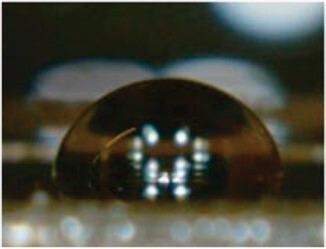
Contact angle (°)	63.1 ± 1.4	66.4 ± 0.8	65.8 ± 0.9	50.4 ± 2.4	76.7 ± 1.7

Data are presented as the mean ± SE of eight or more independent measurements.

### Platelet adhesion

**Figure 2.**
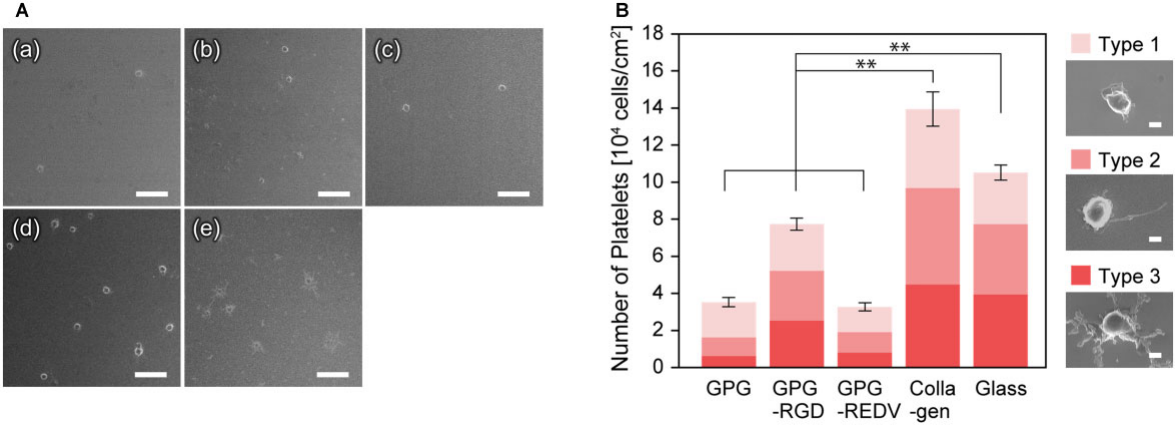
(**A**) FE-SEM Images of adhered platelets on (**a**) GPG, (**b**) GPG-RGD, (**c**) GPG-REDV, (**d**) collagen and (**e**) uncoated coverglass. Bars correspond to 10 μm. (**B**) Number of platelets adhered to each surface. **P <* *0.01. Bars represent 1 μm.

Each surface was incubated with platelet suspensions prepared from human whole blood for one hour at 37°C (Fig. 2A). Lower numbers of platelets were found to adhere to each ELP nanofiber (GPG, GPG-RGD and GPG-REDV) compared to collagen and uncoated glass (Fig. 2B). Fewer platelets adhered to GPG and GPG-REDV compared to GPG-RGD (*P *<* *0.01). Moreover, most activated, Type 3 platelets were rarely observed on GPG and GPG-REDV.

### Endothelial cell adhesion, proliferation and activity

**Figure 3.**
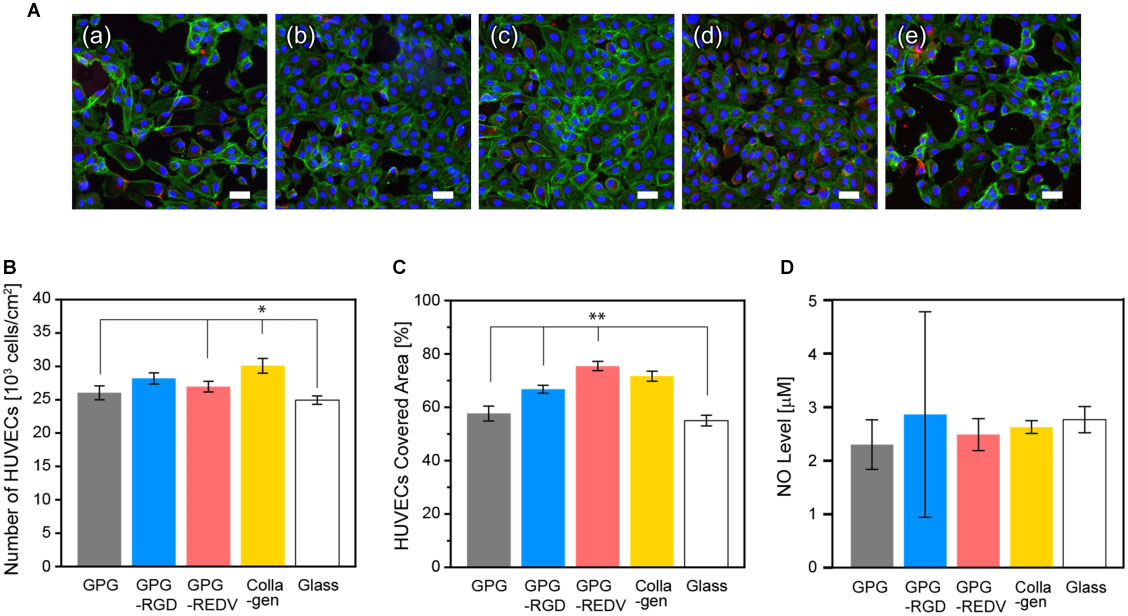
(**A**) Fluorescence microscopy images of HUVECs (blue, nuclei; green, actin; red, vinculin) adhered on (**a**) GPG, (**b**) GPG-RGD, (**c**) GPG-REDV, (**d**) collagen and (**e**) uncoated coverglass after 24 h. Bars correspond to 50 μm. (**B**) Numbers and (**C**) coverage of HUVECs adhered to each surface. *P <* *0.05, **P <* *0.01. (**D**) NO levels in cell culture media.

HUVECs were seeded onto prepared surfaces at a density of 1.0 × 10^4^ cells/well and allowed to adhere for 24 h (Fig. 3A). Cells with flat polygonal or fusiform morphology were observed on all surfaces. Figure 3B and C show cell numbers and coverage, respectively. Collagen had the greatest number of adhered cells compared to other surfaces (collagen vs. GPG, GPG-REDV and glass, *P *<* *0.05 for all). No statistically significant difference in cell numbers was observed between GPG-RGD and collagen. The greatest cell coverage was observed with GPG-REDV (GPG-REDV vs. GPG1, GPG-RGD and glass, *P *<* *0.01 for all), with no statistical difference in cell coverage observed between GPG-REDV and collagen.

To examine the activity of HUVECs, NO release from cultured cells was measured. NO plays significant roles in maintaining vascular hemostasis [51]; promoting EC proliferation [52], inhibiting SMC overactivity [53] and inhibiting platelet aggregation [54]. HUVECs were cultured on each surface (1.0 × 10^4^ cells/well) for 24 h, and the amount of accumulated NO in cell media was estimated (Fig. 3D). NO levels were similar between surfaces after 24 h, with no statistically significant differences observed.

**Figure 4.**
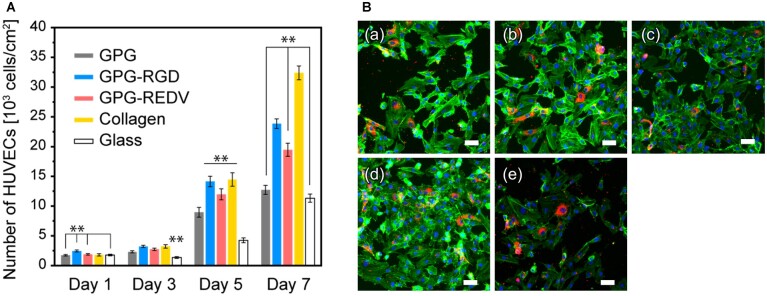
(**A**) Number of HUVECs on each surface. **P <* *0.01. (**B**) Fluorescence microscopy images of HUVECs (blue, nuclei; green, actin; red, vWF) on (**a**) GPG, (**b**) GPG-RGD, (**c**) GPG-REDV, (**d**) collagen and (**e**) uncoated coverglass on Day 7. Bars correspond to 50 μm.

The proliferation of HUVECs on each surface was then examined by seeding cells at a density of 1.0 × 10^3^ cells/well. Overall, cell numbers increased over 7 days for all surfaces (Fig. 4A). On Day 1, the greatest cell numbers were observed on GPG-RGD compared to other surfaces (GPG-RGD vs. GPG, GPG-REDV and glass, *P *<* *0.01 for all; GPG-RGD vs. collagen, *P *<* *0.05). Greater cell numbers were observed on protein-coated surfaces than uncoated glass on Day 3 (protein-coated surfaces vs. glass, *P *<* *0.01). On Day 5, greater cell proliferation was observed on GPG-RGD, GPG-REDV and collagen compared to GPG and glass (*P *<* *0.05). The greatest cell proliferation was observed on collagen, followed by GPG-RGD, GPG-REDV, GPG and glass on Day 7. Cell numbers were significantly higher on GPG-REDV compared to GPG and glass (*P *<* *0.01). Immunostaining of cells on Day 7 demonstrated expression of vWF, a specific marker of endothelial cells, by cells grown on all surfaces, demonstrating maintenance of endothelial cell phenotype by HUVECs (Fig. 4B). However, there were both vWF-positive and negative cells on each surface, and the percentage of vWF-positive cells varied by location.

### Smooth muscle cell adhesion, proliferation and αSMA expression

**Figure 5.**
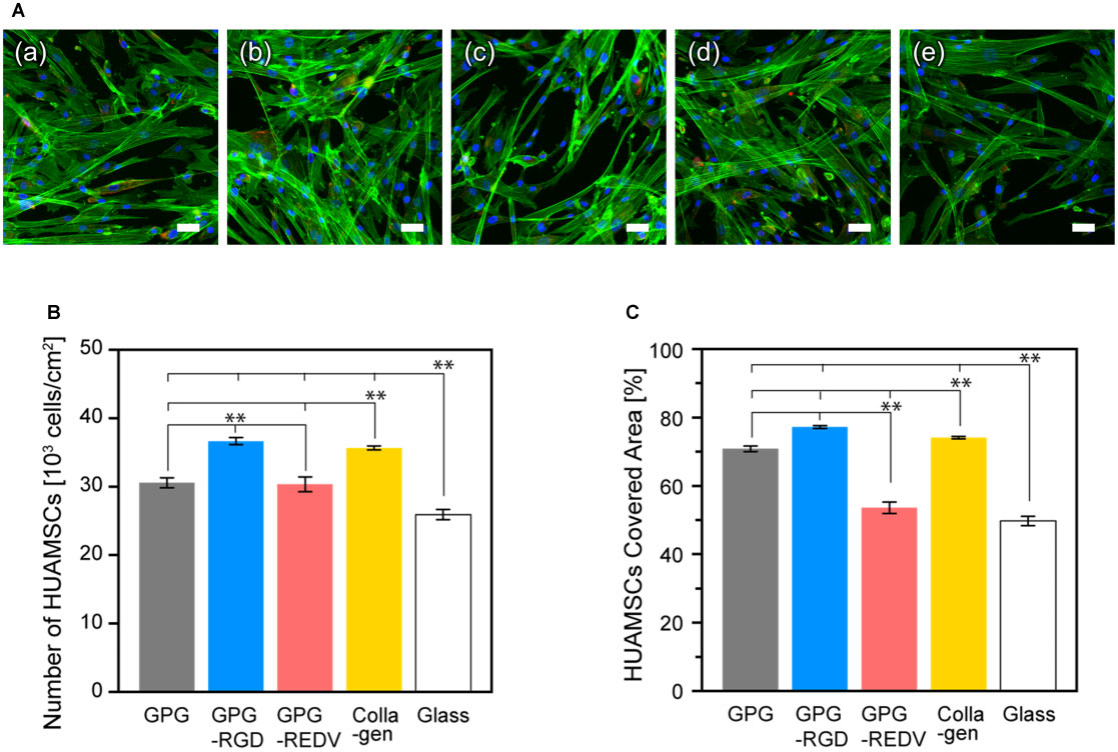
(**A**) Fluorescence microscopy images of HUASMCs (blue, nuclei; green, actin; red, vinculin) adhered on (**a**) GPG, (**b**) GPG-RGD, (**c**) GPG-REDV, (**d**) collagen and (**e**) uncoated coverglass after 24 h. Bars correspond to 50 μm. (**B**) Number and (**C**) coverage of HUASMCs adhered to each surface. **P <* *0.01.

HUASMCs were seeded on prepared surfaces at a cell density of 1.0 × 10^4^ cells/well and allowed to adhere for 24 h (Fig. 5A). Cells with elongated morphology were observed on all surfaces. Cell adhesion was greatest on GPG-RGD and collagen, followed by GPG and GPG-REDV (GPG-RGD and collagen vs. GPG and GPG-REDV; *P *<* *0.01 for all). Cell numbers were significantly lower on glass compared to protein-coated surfaces (glass vs. GPG, GPG-RGD, GPG-REDV and collagen, *P *<* *0.01 for all). GPG-REDV and glass had the lowest cell coverage (Fig. 5B).

**Figure 6.**
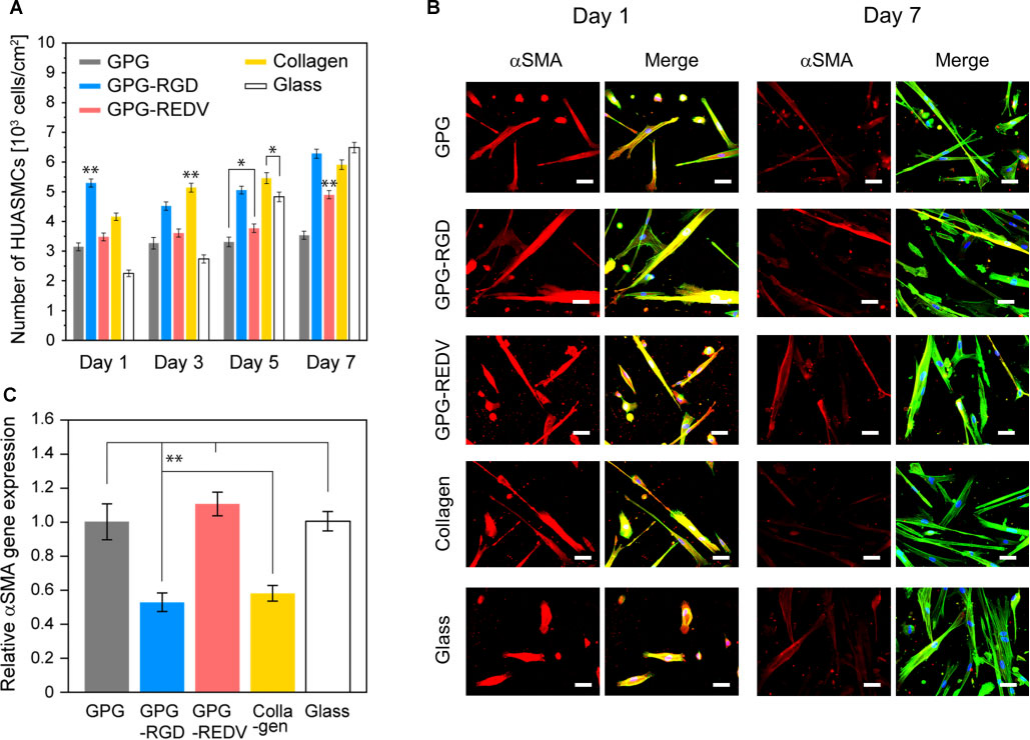
(**A**) Number of HUASMCs on each surface. *P <* *0.05, **P <* *0.01. (**B**) Fluorescence microscopy images of HUASMCs (red, αSMA; blue, nuclei; green, actin). Bars correspond to 50 μm. (**C**) Relative αSMA gene expression of HUASMCs on Day 7. The level of αSMA expression by HUASMCs cultured on glass was set at 1.0. **P <* *0.01.

Figure 6A shows the proliferation of HUASMCs on each surface. Overall, the proliferation of HUASMCs was lower than that observed for HUVECs. The greatest cell proliferation was observed on glass, with a >200% increase in cell number observed from Days 1 to 7. In contrast, minimal cell proliferation was observed on GPG. Moderate cell proliferation was observed on GPG-RGD, GPG-REDV and collagen. Cell numbers were lowest on GPG-REDV (REDV vs. GPG-RGD and collagen, *P *<* *0.01 for both). The phenotype of HUASMCs was then assessed using immunostaining (Fig. 6B) and qRT-PCR (Fig. 6C). αSMA is a contractile protein used as an early marker of the smooth muscle lineage during differentiation [55]. αSMA was expressed by cells on all surfaces on Day 1 (Fig. 6B). The fluorescence intensity of αSMA staining decreased on Day 7, particularly for cells cultured on collagen. The αSMA gene expression by cells was higher on GPG, GPG-REDV and glass than GPG-RGD and collagen on Day 7 (Fig. 6C).

## Discussion

The aim of the present study was to evaluate the biological characteristics of GPG nanofibers, which are formed from a double-hydrophobic type of ELP [44–49], for use in TEVGs. As important requirements for TEVGs include: (i) anti-thrombogenicity; (ii) early endothelialization; and (iii) prevention of over-activation of SMCs, we designed and fabricated a new derivative, termed GPG-REDV, which appears to interact exclusively with ECs while maintaining the biological characteristics of elastin. In the present study, we compared GPG-REDV to GPG with no additional functional sequences, GPG comprising an RGD sequence, collagen and cell culture glass.

GPG-REDV was shown to self-assemble into nanofibers in water, as previously reported for GPG and GPG-RGD [48]. We then coated borosilicate glass surfaces with GPGs and collagen for cell culture. FE-SEM imaging demonstrated the disappearance of glass particulate protrusions after coating, indicating the presence of proteins on the glass surface (Fig. 1). Water contact angles decreased in the order of glass (76.7 ± 1.7°) > GPG-RGD (66.4 ± 0.8°) ∼ GPG-REDV (65.8 ± 0.9°) ∼ GPG (63.1 ± 1.4°) > collagen (50.4 ± 2.4°), indicating that the surfaces were more hydrophilic after protein coatings (Table 2). GPGs are relatively hydrophobic compared to collagen as they are rich in hydrophobic amino acids (Table 1). However, GPGs remain more hydrophilic than glass, likely due to exposure of the hydrophilic C-termini (His-tag and added sequences) of nanofibers as a result of their amphiphilic design.

Platelet adhesion assays are the most direct method of evaluating the thrombogenicity of material surfaces. After contact with platelet suspensions for 1 h, GPGs had significantly lower numbers of adhered platelets compared to collagen and glass (Fig. 2). This result indicates that GPG nanofibers keep the anti-thrombogenic properties of natural elastin. Among GPGs, GPG and GPG-REDV had the lowest number of adhered platelets. Platelets adhered to GPGs were predominantly Type 1, the most inactive type, indicating physical adsorption. A higher number of platelets with a lower proportion of Type 1 platelets was observed on GPG-RGD in-keeping with the recognition of RGD by platelet integrins, including α_IIb_β_3_, α_v_β_3_ and α_5_β_1_ [56]. Collagen has the greatest number of adhered platelets as collagen contains GXX'GER motifs that bind to integrin α_2_β_1_ of platelets [57], and platelets express the collagen receptors glycoprotein VI [58]. The number of platelets adhered to glass was intermediate between collagen and GPG-RGD despite the absence of biological cues. This finding may be attributed to the greater hydrophobicity of glass. Lee and Lee [59] reported platelet adhesion onto surfaces across a wettability gradient. Platelet adhesion increased with increasing surface hydrophobicity, which was attributed to plasma protein adsorption on the surface, indicating a lower concentration of plasma proteins that inhibit platelet adhesion are adsorbed by more hydrophobic surfaces. Platelet adhesion on the hydrophobic surface is also explained by the conformational change of the surface-adsorbed fibrinogen (Fg), a plasma glycoprotein that is involved in blood coagulation. Sivaraman *et al.* [60] reported that platelet adhesion was strongly correlated with the degree of adsorption-induced unfolding of Fg that was caused on more hydrophobic surfaces.

In the present study, the number of adhered HUVECs was greater on collagen and GPG-RGD after 24 h (Fig. 3B), while greater cell coverage was observed on GPG-REDV and collagen (Fig. 3C). Integrin α_4_β_1_-mediated actin fiber extension may occur on GPG-REDV. The greatest proliferation of HUVECs was observed on collagen followed by GPG-RGD and then GPG-REDV. However, when comparing cell numbers between Days 1 and 7, there was a 9.7-fold increase in cell numbers on GPG-RGD and a 10.3-fold increase on GPG-REDV. Thus, the difference in the number of cells on GPG-RGD and GPG-REDV on Day 7 appears to be due to the difference in the number of adherent cells on Day 1. Maintenance of endothelial phenotype was suggested by NO release (Fig. 3D) and vWF expression (Fig. 4B) on all surfaces. However, further studies including the elucidation of cell–cell interactions are needed to explain the presence of both vWF-positive and negative cells with the varied percentage by location.

Vascular SMCs have a spindle-shaped contractile phenotype in normal tissues. Contractile SMCs are in the G0/G1 phase of the cell cycle and express high levels of markers such as αSMA and calponin, which contribute to vascular contractility [61]. However, vascular SMCs subjected to external stress from platelets and macrophages undergo reversible transformation to the synthetic phenotype. Synthetic SMCs have high migration, proliferation and extracellular matrix production. At the time of vascular grafting, overgrowth of synthetic SMCs may cause neointimal hyperplasia resulting in vessel occlusion [62]. Accordingly, it is necessary to maintain the phenotype of contractile SMCs and prevent the hyper-proliferation of SMCs in artificial vascular materials.

Greater numbers of HUASMCs were adhered to GPG-RGD and collagen after 24 h, indicating that GPG-RGD and collagen were recognized by integrins of the HUASMCs (Fig. 5). Cells were more adherent to GPG and GPG-REDV compared to glass. Other than the marked difference observed between GPG and GPG-REDV, cell coverage corresponds to the number of adhesions (Fig. 5C). The HUASMCs might adhere better to hydrophilic surfaces as previously reported [62]; the surface wettability is GPG ∼ GPG-REDV > glass. However, wettability alone is unable to explain the greater cell coverage observed with GPG compared to GPG-REDV. Karnik *et al.* [35] reported that elastin stimulates actin stress fiber formation via G protein-coupled signaling pathways in response to direct interactions between the receptor complex named elastin binding protein and the VGVAPG domain of tropoelastin. This pathway may be activated by GPGs via VPGVG repeat motifs, which are more exposed on the nanofiber surface on GPG compared to GPG-REDV as GPG has no additional functional sequences at the C-terminus. Further studies are required to evaluate this hypothesis.

The proliferation of HUASMCs was slower than HUVECs on all surfaces (Figs 4A and 6A). The numbers of HUASMCs on Day 1 demonstrated a similar trend to that of cell adhesion after 24 h (Fig. 5A). The increase in cell numbers on Day 7 compared to Day 1 was particularly high for glass (288%) compared to collagen (142%), GPG-REDV (141%), GPG-RGD (119%) and GPG (112%). Ligands present on proteins (integrin-binding ligands or VPGVG) appear to increase the number of initial cell adhesions but inhibit subsequent proliferation. With particular attention to GPG-RGD and collagen, integrin-binding ligands present on protein surfaces appear to cause a marked increase in the number of initial cell adhesions and a decrease in αSMA gene expression during long-term cell culture (Figs 5A and 6C). We observed no clear relationship between cell proliferation and αSMA gene expression on Day 7. Longer-term cell culture experiments may be required to fully determine the relationship between cell proliferation and αSMA gene expression.

Finally, the biological characteristics of the materials evaluated in the present study are summarized in Table 3. A plus sign indicates the characteristics are preferable for applications in TEVG. Overall, GPG-REDV was considered a suitable material due to excellent platelet inhibition, relatively higher EC adhesion and proliferation characteristics, and ability to prevent SMC over-activity.

**Table 3.** Biological characteristics of materials evaluated in the present study

Protein	Platelet inhibition^a^	EC activation			SMC maintenance	
		Adhesion	Proliferation	NO release	Proliferation inhibition	αSMA expression
GPG	+				+	
GPG-RGD	+	+	+		+	−
GPG-REDV	+	+	+		+	
Collagen	−	+	+		+	−

Evaluations were made by comparison with cell culture glass. +, superior to glass; −, inferior to glass. Blank fields indicate equivalence to glass.

a +, platelet count less than glass; −, platelet count greater than glass.

## Conclusions

In this article, we strategically designed a GPG-REDV polypeptide with an endothelial cell adhesion sequence added to an elastin-like sequence with fiber-forming ability. The resultant nanofibers showed the biological characteristics including inhibition of platelet adhesion, activation of endothelial cells for adhesion and proliferation, and maintenance of SMCs in a contractile phenotype to prevent them from over-activation. In addition to the findings of this study, control of the mechanical properties and biodegradability of the material is necessary to realize small-diameter vascular graft with long patency. The challenges may be overcome by fabricating organized elastin-like structures using the present material in future studies.

## Supplementary Material

rbac111_Supplementary_Data
