# Supplementary material for: Biological properties of self-assembled nanofibers of elastin-like block polypeptides for tissue-engineered vascular grafts: platelet inhibition, endothelial cell activation and smooth muscle cell maintenance
Source: Regen Biomater. 2022 Dec 28;10:rbac111. doi: 10.1093/rb/rbac111 (PMC9845521; doi:10.1093/rb/rbac111)
Supplement: rbac111_Supplementary_Data [file rbac111_supplementary_data.docx]

Supplementary Data

**Biological properties of self-assembled nanofibers of elastin-like block polypeptides for tissue-engineered vascular grafts: Platelet inhibition, endothelial cell activation, and smooth muscle cell maintenance**

Kazuki Natsume^1^, Jin Nakamura^2^, Kazuhide Sato^3,4^, Chikara Ohtsuki^1^, Ayae Sugawara-Narutaki^*5^

^1^Department of Materials Chemistry, Graduate School of Engineering, Nagoya University, Furo-cho, Chikusa-ku, Nagoya 464-8603, Japan.

^2^*Department of Biological Functions Engineering,* Graduate School of Life Science and Systems Engineering, Kyushu Institute of Technology, 2-4 Hibikino, Wakamatsu-ku, Kitakyushu-shi, Fukuoka 808-0196, Japan.

^3^Institute for Advanced Research, Nagoya University, Furo-cho, Chikusa-ku, Nagoya 464-8601, Japan.

^4^Department of Respiratory Medicine, Graduate School of Medicine, Nagoya University, Tsurumai-cho, Showa-ku, Nagoya 466-8560, Japan.

^5^Department of Energy Engineering, Graduate School of Engineering, Nagoya University, Furo-cho, Chikusa-ku, Nagoya 464-8603, Japan

**Correspondence to:** A. Sugawara-Narutaki; TEX/FAX: +81-52-789-3602; email: ayae@energy.nagoya-u.ac.jp

1. **Construction of pET22b(+)-REDV plasmid**

The plasmid pET22b(+)-REDV encoding GPG-REDV was constructed by inverse polymerase chain reaction (iPCR)-based site-directed mutagenesis using pET22b(+)-GPG3 as the template.^1^ In this method, gene sequence encoding GRGDS in pET22b(+)-GPG3 was converted into GREDV in pET22b(+)-REDV. The primers used for this purpose were purchased from Life Technologies Japan Ltd. The iPCR was performed using KOD-Plus Mutagenesis Kit (Toyobo, Japan) according to the manufacturer’s protocol. After iPCR, the template plasmid was digested using a restriction enzyme DpnI (Toyobo, Japan). The iPCR product remained was self-ligated in the presence of T4 Polynucleotide Kinase (Toyobo, Japan) and Ligation High (Toyobo, Japan) to generate pET22b(+)-REDV. It was then transformed into *E. coli* strain SURE2 competent cells (Agilent Technologies, USA). After cell culture, the plasmid was purified using QIAprep Spin Miniprep Kit (Qiagen, Germany). The formation of correct plasmid was confirmed using sequencing analysis (Center for Gene Research, Nagoya University).

1. **Synthesis and Purification of GPG-REDV**

Protein purification was conducted by metal affinity chromatography as previously reported^2^ and confirmed by sodium dodecyl sulfate polyacrylamide gel electrophoresis (SDS-PAGE) and matrix-assisted laser desorption ionization-time of flight mass spectroscopy (MALDI-TOF-MS). MALDI-TOF-MS was performed using an AXIMA-CFR Plus spectrometer (Shimadzu Corporation, Japan).

The calculated molecular weight of GPG-REDV is 17,736 Da. In the image of SDS-PAGE (Figure S1a), a thick protein band was observed around the corresponding molecular weight position. There also was a thin band at 25 kDa, which was presumably attributed to SlyD (molecular weight: 20.8 kDa), a histidine-rich protein derived from *E.coli*.^3^ MALDI-TOF-MS showed the presence of a peak at 17,797.5 Da (Figure S1b). The peak originating from SlyD was not detected in the MS spectrum. These results suggest that GPG-REDV was successfully expressed and purified as the main product.


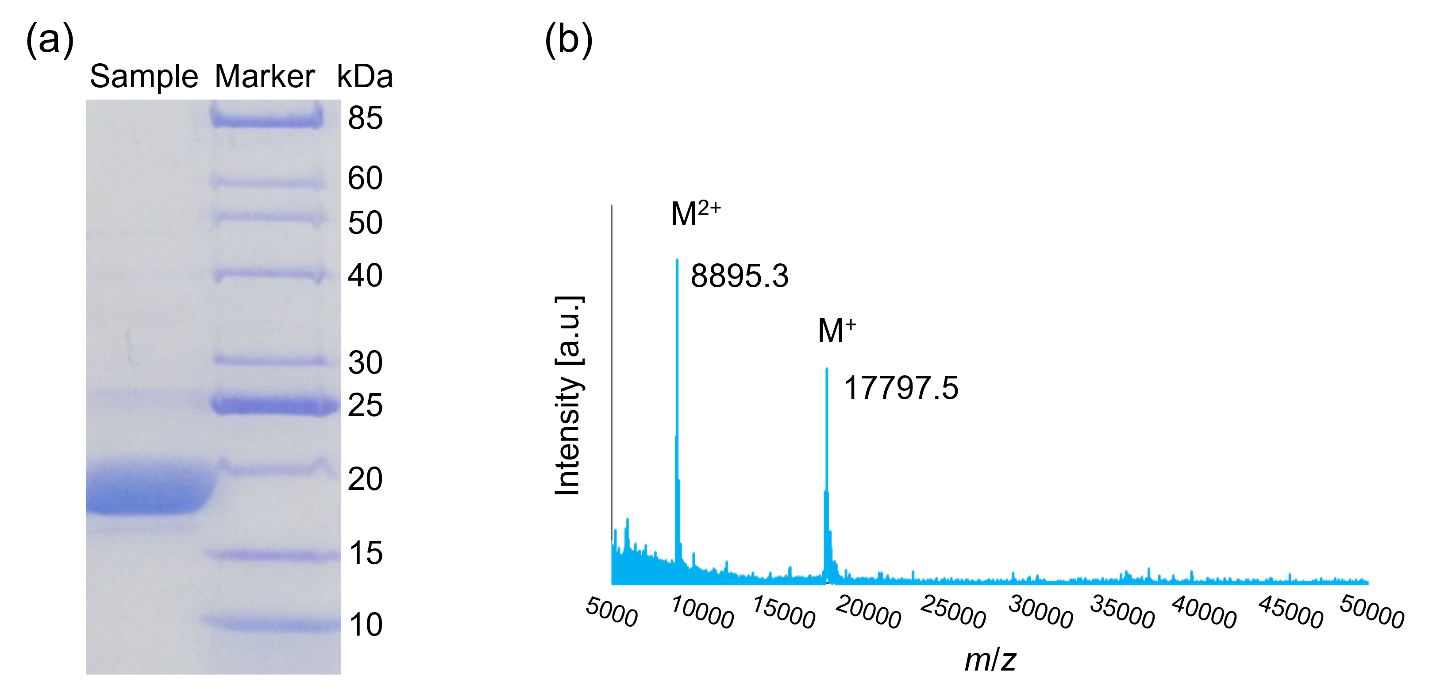


**Figure S1** The results of (a) SDS-PAGE and (b) MALDI-TOF-MS of GPG-REDV.

1. **Nanofiber formation from GPG-REDV**

The lyophilized powder of GPG-REDV was dissolved in water at 4ºC to a final concentration of 20 μM (0.35 mg/mL). The solution was incubated at 37 °C for a week in an incubator. The sample (20 μL) was drop cast on a mica substrate (Nilaco, Japan) and allowed to dry at 37 °C. Atomic force microscopy (AFM) was performed with a MFP-3D Origin^TM^ AFM (Oxford Instruments, UK) in a tapping mode using OMCL-AC240TS (Olympus, Japan) as a cantilever.

Nanofibers with bead-on-string structures were observed in AFM (Figure S2a). The diameter of nanofibers is less than 50 nm at the thinnest part. The beaded morphology is the general characteristic of GPGs including GPG without the ligand for integrin and GPG-RGD.^1^

To further confirm the self-assembling nature of GPG-REDV, gelation test was conducted. The aqueous solution of GPG-REDV (5 mg/mL) was gelled after incubation at 45°C for a day. The gelling ability is also the characteristic of a series of GPG derivatives.^4^


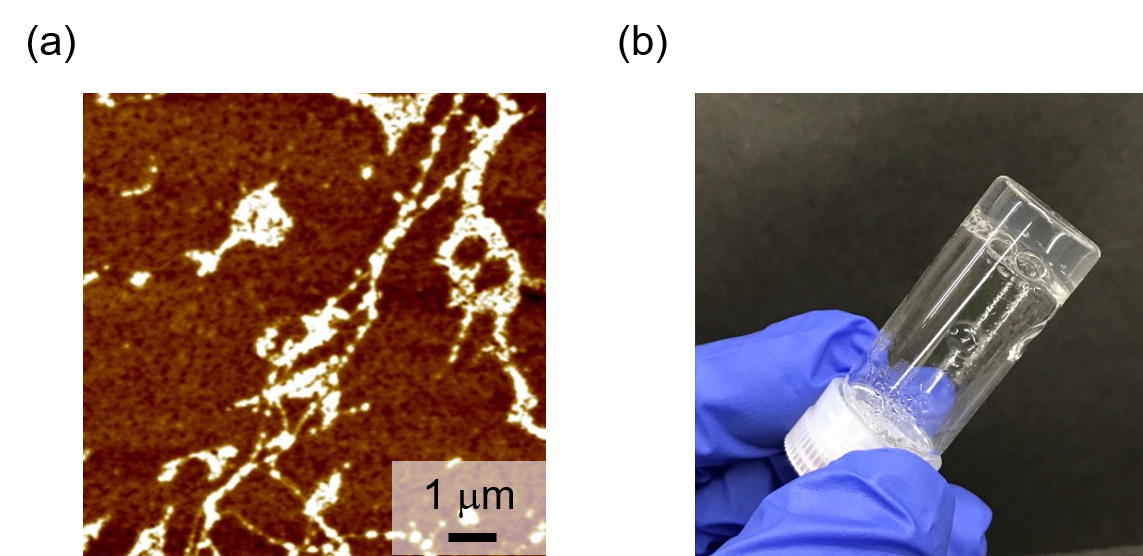


**Figure S2** (a) AFM image of GPG-REDV nanofibers and (b) photo of the hydrogel of GPG-REDV.

**References**

1. Le DHT, Tsutsui Y, Sugawara-Narutaki A, Yukawa H, Baba Y, Ohtsuki C. Double-hydrophobic elastin-like polypeptides with added functional motifs: self-assembly and cytocompatibility. *J Biomed Mater Res A* 2017;105:2475–84.
2. Le DHT, Hanamura R, Pham DH, Kato M, Tirrell DA, Okubo T, Sugawara-Narutaki A. Self-assembly of elastin-mimetic double hydrophobic polypeptides. *Biomacromolecules* 2013;14:1028–34.
3. Mokhonov VV, Vasilenko EA, Gorshkova EN, Astrakhantseva IV, Novikov DV, Novikov VV. SlyD-deficient Escherichia coli strains: a highway to contaminant-free protein extraction. *Biochem Biophys Res Commun* 2018;499:967–72.
4. Sugioka Y, Nakamura J, Ohtsuki C, Sugawara-Narutaki A. Thixotropic hydrogels composed of self-assembled nanofibers of double-hydrophobic elastin-like block polypeptides. Int J Mol Sci 2021;22:4104.
